# Unveiling preeclampsia: diagnostic value and potential molecular mechanisms of abnormally methylated immune-related genes

**DOI:** 10.1590/1414-431X2025e15317

**Published:** 2026-03-09

**Authors:** Yuan Yuan, Qin Wang, Xiao Su, Lu Zhong

**Affiliations:** 1 Department of Gynecology and Obstetrics, The 3rd Affiliated Hospital of Chengdu Medical College, Pidu District People's Hospital, Chengdu, China

**Keywords:** Preeclampsia, Genes, Immune, DNA methylation, Classification model

## Abstract

Preeclampsia (PE) is a life-threatening obstetric complication, and DNA methylation and immune system disorders play a key role in its development. This study aimed to explore the potential mechanisms and values of abnormally methylated immune-related differentially expressed genes (DEGs) in PE. Gene expression profiles and methylation data of PE were downloaded from the Gene Expression Omnibus (GEO) database. Immune-related genes were downloaded from the ImmPort database. Subsequently, differential expression analysis, functional annotation, immune cell infiltration analysis, Pearson correlation analysis, construction of classification models and miRNA-mRNA interaction network, and real-time PCR validation were carried out. Ten key abnormally methylated immune-related DEGs (*ESRRG*, *FGF10*, *AHNAK*, *STC2*, *PPARG*, *LTF*, *MX1*, *ESR1*, *RELB*, and *JAG2*) were identified and may be potential diagnostic biomarkers for PE. The decision tree (DT), random forests (RF), and support vector machine (SVM) classification models constructed based on these 10 genes exhibited a certain level of diagnostic accuracy. Compared with a single DEG, these classification models may have relatively higher diagnostic reference value. Functional annotation results showed that rap1, PI3K-Akt, and calcium signaling pathways may play a regulatory role in PE. The infiltration levels of monocytes, M2 macrophages, neutrophils, Tregs, and eosinophils in the PE group were abnormal. Key abnormally methylated immune-related DEGs were significantly correlated with the infiltration levels of immune cells. Moreover, 6 miRNA-mRNA pairs (hsa-miR-181b-5p-ESR1, hsa-miR-152-3p-ESR1, hsa-miR-26b-3p-ESR1, hsa-miR-4672-ESRRG, hsa-miR-502-3p-AHNAK, and hsa-miR-3059-5p-STC2) were identified. Key abnormally methylated immune-related DEGs may be associated with the immune mechanism of PE, given their correlation with related signaling pathways and immune cell infiltration.

## Introduction

Preeclampsia (PE) is a pregnancy-specific syndrome that remains a leading cause of maternal and perinatal morbidity and mortality worldwide (1). PE is characterized by new-onset hypertension and proteinuria after 20 weeks of pregnancy and can also present with a wide range of other symptoms, including edema, thrombocytopenia, impaired liver function, and renal insufficiency. The condition not only poses significant risks to the mother's health, such as an increased likelihood of developing cardiovascular diseases later in life (2), but also has profound impacts on the fetus, leading to fetal growth restriction, preterm birth, and even stillbirth.

Despite extensive research, the exact pathophysiology of PE remains incompletely understood. The current “two-stage” hypothesis proposes that abnormal trophoblast invasion and inadequate remodeling of the spiral arteries in the first stage result in placental ischemia and hypoxia. This, in turn, triggers the release of various factors into the maternal circulation in the second stage, leading to systemic endothelial dysfunction, inflammation, and oxidative stress (3). Among these complex pathogenic mechanisms, both immune dysregulation and abnormal DNA methylation have emerged as key players in the development of PE. In the context of the immune system, pregnancy is a unique immune state, where the mother's immune system must tolerate a semi-allogeneic fetus. In normal pregnancy, the delicate balance of the immune response is maintained at the maternal-fetal interface. However, in PE, mounting evidence supports immune system dysregulation (4,5). Therefore, the identification of immune-related molecular biomarkers in PE is helpful to understand the molecular mechanism of the disease and provide potential diagnostic and therapeutic targets.

DNA methylation is an epigenetic modification that plays a crucial role in gene expression regulation. In the context of pregnancy, DNA methylation changes in the placenta and maternal blood are involved in the pathogenesis of PE (6). Some genes related to trophoblast function, such as those involved in cell invasion and angiogenesis, show abnormally methylated levels. These changes may disrupt the normal biological functions of trophoblasts, leading to abnormal placental development and contributing to the onset of PE (7). Therefore, exploring the DNA methylation modification data of genes is vital to illuminating the epigenetic regulatory mechanisms of PE.

In disease diagnosis and prediction, accurate and timely identification of disease patterns is of vital importance. Early and accurate diagnosis not only buys patients precious treatment time, but also significantly improves treatment outcomes and prognosis. With the rapid advancements in technology, machine learning has emerged as a powerful tool in medical research. Machine learning algorithms, including random forests (RF), decision tree (DT), and support vector machine (SVM), have been used to classify outcomes in biomedical datasets (8). The receiver operating characteristic (ROC) curve serves as a reliable metric for assessing the performance of diagnostic tools. In ROC analysis, a greater area under the curve (AUC) indicates enhanced diagnostic accuracy (9). In this study, abnormally methylated immune-related differentially expressed genes (DEGs) in PE were identified based on the Gene Expression Omnibus (GEO, https://www.ncbi.nlm.nih.gov/geo/) database, and RF, DT, and SVM classification models were constructed. This study aimed to explore the potential mechanisms and values of abnormally methylated immune-related DEGs in PE through differential analysis, functional annotation, immune correlation analysis, construction of classification models, and establishment of mRNA-miRNA interaction networks.

## Material and Methods

### Data source

PE-related datasets in the GEO database based on the keyword “(preeclampsia) AND ‘Homo sapiens'[porgn:__txid9606]” were retrieved. The search was limited by study type using “expression profiling by array” and “methylation profiling by array”. Subsequently, the datasets that met the following criteria were included in this study: 1) must be mRNA transcriptome data and DNA methylation data of the whole genome; 2) from PE and control placental tissue samples; 3) standardization or the original dataset; 4) being from the same year. According to the above screening criteria, 1 mRNA dataset (GSE75010) and 1 methylation dataset (GSE75196) were obtained (Supplementary Table S1). For the GSE75010 dataset, the original creators of the dataset employed empirical Bayes methods for normalization and batch correction (10). For the GSE75196 dataset, the original creators of the dataset normalized the data using the dasen function (11).

### Identification of DEGs and abnormally methylated genes

The GSE75010 dataset was downloaded from the GEO database, and the probe was mapped to the gene. The average value of a gene from multiple probes was taken as the expression level of the gene. The limma package in R software (version 4.0.5) was used for differential expression analysis to identify DEGs. The screening criterion was adj.P<0.05. The GSE75196 dataset was downloaded from the GEO database. The CHAMP package in R software (version 4.0.5) was used for differential methylation analysis to identify differential methylation sites and thereby screen out differentially methylated genes. The screening criteria were P<0.05 and |delta Beta| >0.1.

### Functional annotation of DEGs and abnormally methylated genes

To understand the biological processes involved in DEGs and abnormally methylated genes, Gene Ontology (GO) and Kyoto Encyclopedia of Genes and Genomes (KEGG) functional enrichment analyses were performed using the DAVID database (https://david.ncifcrf.gov/tools.jsp). KEGG is an important database in the field of bioinformatics that analyzes gene function by integrating genomic information with higher order functional information. GO is a resource that supplies information about gene product function using ontologies to represent biological knowledge. These ontologies include molecular function (MF), biological process (BP), and cellular components (CC). The screening criterion was P<0.05.

### Identification of abnormally methylated immune-related DEGs

A total of 1793 immune-related genes were downloaded from the ImmPort database (https://www.immport.org/shared/home). The intersection of DEGs, abnormally methylated genes, and immune-related genes was performed to obtain abnormally methylated immune-related DEGs.

### Immune cell infiltration and abnormally methylated immune-related DEGs

The proportion of immune cells in each sample was calculated using the CIBERSORT package in R software (version 4.0.5), and the scores of immune cells in all samples were obtained. The scores of immune cells were sorted into the immune cell infiltration matrix. Subsequently, the Wilcoxon test was used to statistically analyze the difference of immune cell infiltration level between PE and control groups. Pearson correlation analysis was used to analyze the correlation between differential infiltrating immune cells and abnormally methylated immune-related DEGs.

### Identification of key abnormally methylated immune-related DEGs based on machine learning

The importance of abnormally methylated immune-related DEGs was sorted from large to small according to “mean decrease accuracy” value. According to the sorting order, one abnormally methylated immune-related DEG was added from top to bottom. Then, RF algorithm was employed for classification. The accuracy and the AUC were obtained through 10-fold cross-validation, which was used to avoid overfitting (12,13). Then, the key abnormally methylated immune-related DEGs were selected and the DT, RF, and SVM classification models were constructed using the rpart (https://cran.r-project.org/web/packages/rpart/), random forests (https://cran.r-project.org/web/packages/randomForest/), and e1071 (https://cran.r-project.org/web/packages/e1071/index.html) packages in R software (version 4.0.5). The diagnostic ability of the classification models was evaluated through the ROC curve. In addition, ROC analysis of key abnormally methylated immune-related DEGs was performed using the pROC package in R software (version 4.0.5). The AUC was used to evaluate the diagnostic accuracy (9).

### Identification of miRNAs targeting key abnormally methylated immune-related DEGs

Predicted miRNAs targeting key abnormally methylated immune-related DEGs were based on the TargetScan (http://www.targetscan.org/vert_72/) and miRDB (http://mirdb.org/) databases. The GSE206988 dataset was downloaded from the GEO database. Subsequently, the differential expressions of miRNAs in the GSE206988 dataset were analyzed. The screening criterion was P<0.05. The predicted miRNAs and differentially expressed miRNAs (DEmiRNA) were intersected to obtain negatively regulated miRNA-mRNA targeting pairs. Cytoscape (www.cytoscape.org/) was used to visualize the miRNA-mRNA network.

### Real-time PCR validation

The diagnostic criteria for PE were systolic blood pressure ≥140 mmHg and diastolic blood pressure ≥90 mmHg occurring after 20 weeks of gestation, accompanied by urine protein ≥0.3 g/24 h or random urine protein ≥ (++). In addition, PE patients included in this study had no primary diseases of the cardiovascular, cerebrovascular, hepatic, renal, and hematopoietic systems, no history of mental disorders, and had not taken any medications within the previous 3 months. The normal control group comprised healthy individuals matched for age and gender to the PE group. A total of 21 placental tissue samples were included in this study, of which 9 were PE samples and 12 were control samples. TRIzol kit was used to extract total RNA from the samples. FastQuant cDNA first strand synthesis kit (China) was used for reverse transcription. SuperReal PreMix Plus (SYBR Green) kit (China) and Gene-9660 fluorescence quantitative PCR system (China) were used for real-time PCR. The 2^-△△CT^ method was used for the relative quantitative analysis of data.

This study was approved by the Ethics Committee of the Pidu District People's Hospital (202581). This study complied with the Declaration of Helsinki. Written informed consent was obtained from all participants.

## Results

### Identification and functional annotation of DEGs

**Figure 1:**
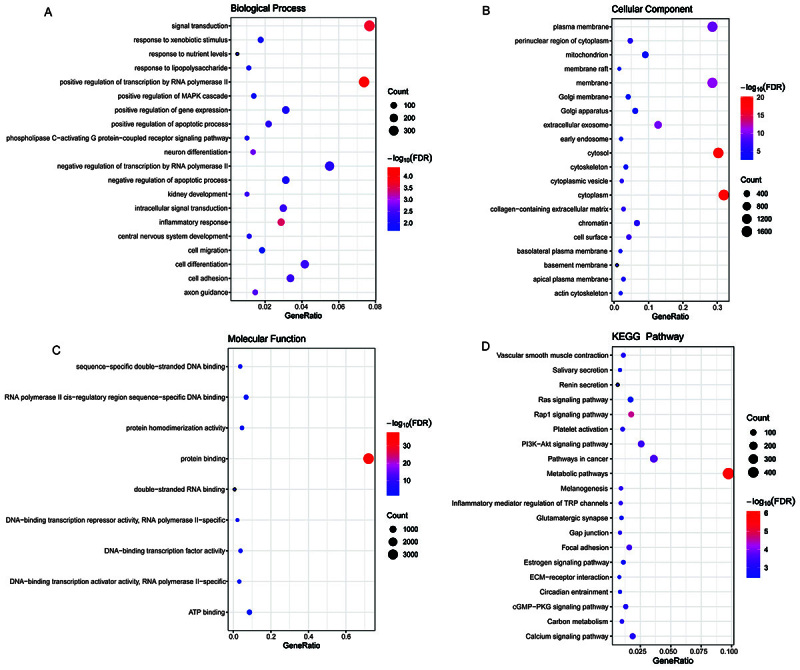
Enrichment analysis of Gene Ontology (GO) and Kyoto Encyclopedia of Genes and Genomes (KEGG) for differentially expressed genes (DEGs). **A**, biological process (BP), **B**, cellular component (CC), and **C**, molecular function (MF) terms enriched in the GO analysis of DEGs. **D**, KEGG analysis of DEGs. The size of each bubble represents the number of enriched genes. The color of bubbles represents enrichment significance. The closer the color is to red, the higher the statistical significance.

Compared with the control group, 2605 up-regulated DEGs and 2524 down-regulated DEGs were identified in the PE group (Supplementary Figure S1A and B). Subsequently, GO and KEGG analyses were performed to understand the potential biological functions of DEGs. In the GO:BP terms, the DEGs were significantly enriched in positive regulation of transcription by RNA polymerase II, signal transduction, and inflammatory response (Figure 1A). In the GO:CC terms, the DEGs were significantly enriched in cytoplasm, cytosol, and membrane (Figure 1B). In the GO:MF terms, the DEGs were significantly enriched in protein binding, DNA-binding transcription repressor activity, RNA polymerase II-specific, and sequence-specific double-stranded DNA binding (Figure 1C). The KEGG results showed that the DEGs were significantly enriched in metabolic pathways, rap1 signaling pathway, and PI3K-Akt signaling pathway (Figure 1D).

### Identification and functional annotation of abnormally methylated genes

**Figure 2:**
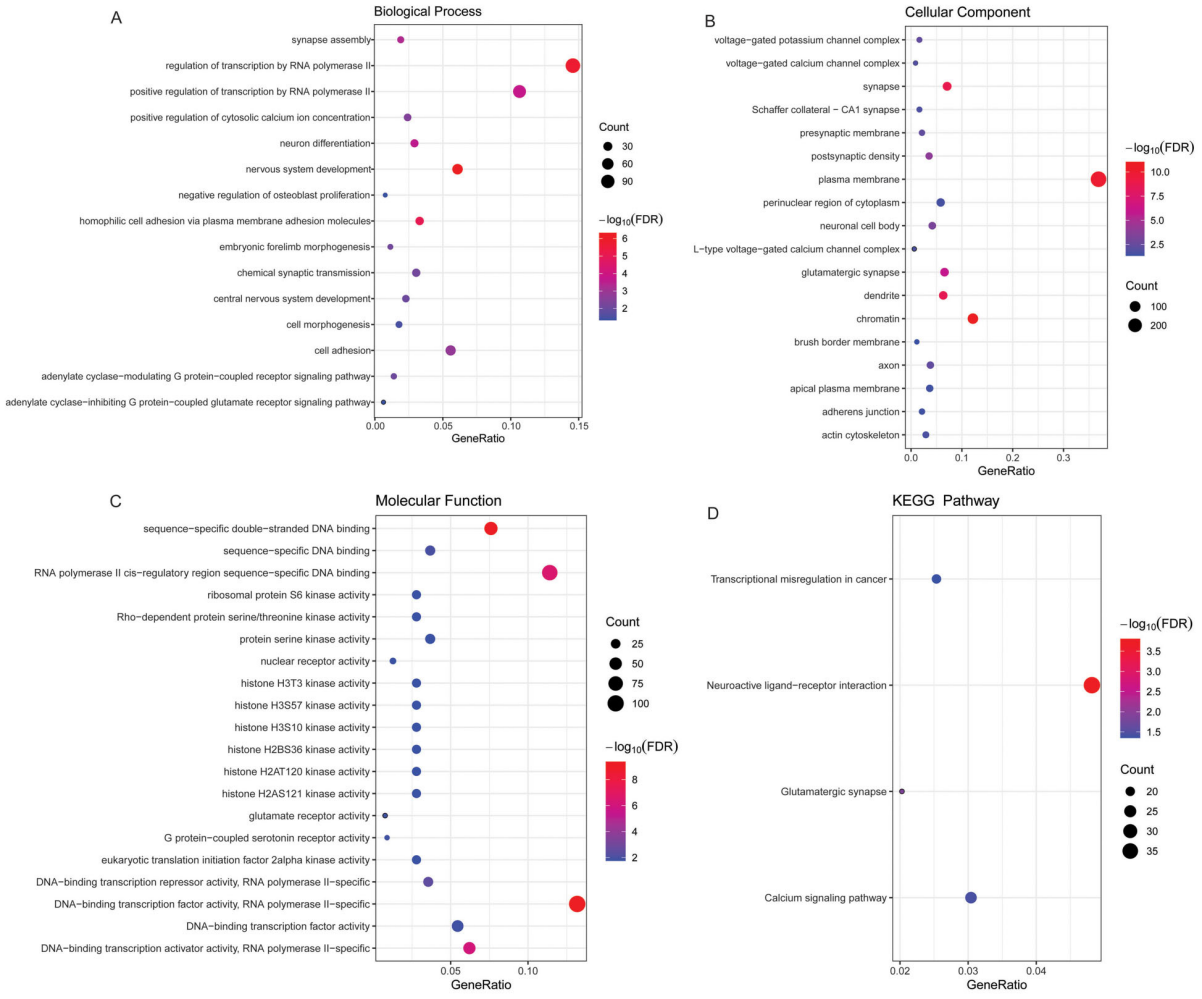
Enrichment analysis of Gene Ontology (GO) and Kyoto Encyclopedia of Genes and Genomes (KEGG) for abnormally methylated genes. **A**, biological process (BP), **B**, cellular component (CC), and **C**, molecular function (MF) terms enriched in the GO analysis of abnormally methylated genes; **D**, KEGG analysis of abnormally methylated genes. The size of each bubble represents the number of enriched genes. The color of bubbles represents enrichment significance. The closer the color is to red, the higher the statistical significance.

Compared with the control group, 1359 differential methylation sites were identified in the PE group. A total of 894 abnormally methylated genes (DMGs) (385 hypermethylated genes and 509 hypomethylated genes) were included in these differential methylation sites (Supplementary Figure S2A and B). Subsequently, GO and KEGG analyses were performed to understand the potential biological functions of abnormally methylated genes. In the GO:BP terms, the abnormally methylated genes were significantly enriched in nervous system development, regulation of transcription by RNA polymerase II, and homophilic cell adhesion via plasma membrane adhesion molecules (Figure 2A). In the GO:CC terms, the abnormally methylated genes were significantly enriched in chromatin, plasma membrane, and synapse (Figure 2B). In the GO:MF terms, the abnormally methylated genes were significantly enriched in DNA-binding transcription factor activity, RNA polymerase II-specific, sequence-specific double-stranded DNA binding, and RNA polymerase II cis-regulatory region sequence-specific DNA binding (Figure 2C). The KEGG results showed that the abnormally methylated genes were significantly enriched in neuroactive ligand-receptor interaction, glutamatergic synapse, and calcium signaling pathway (Figure 2D).

### Identification of abnormally methylated immune-related DEGs

**Figure 3:**
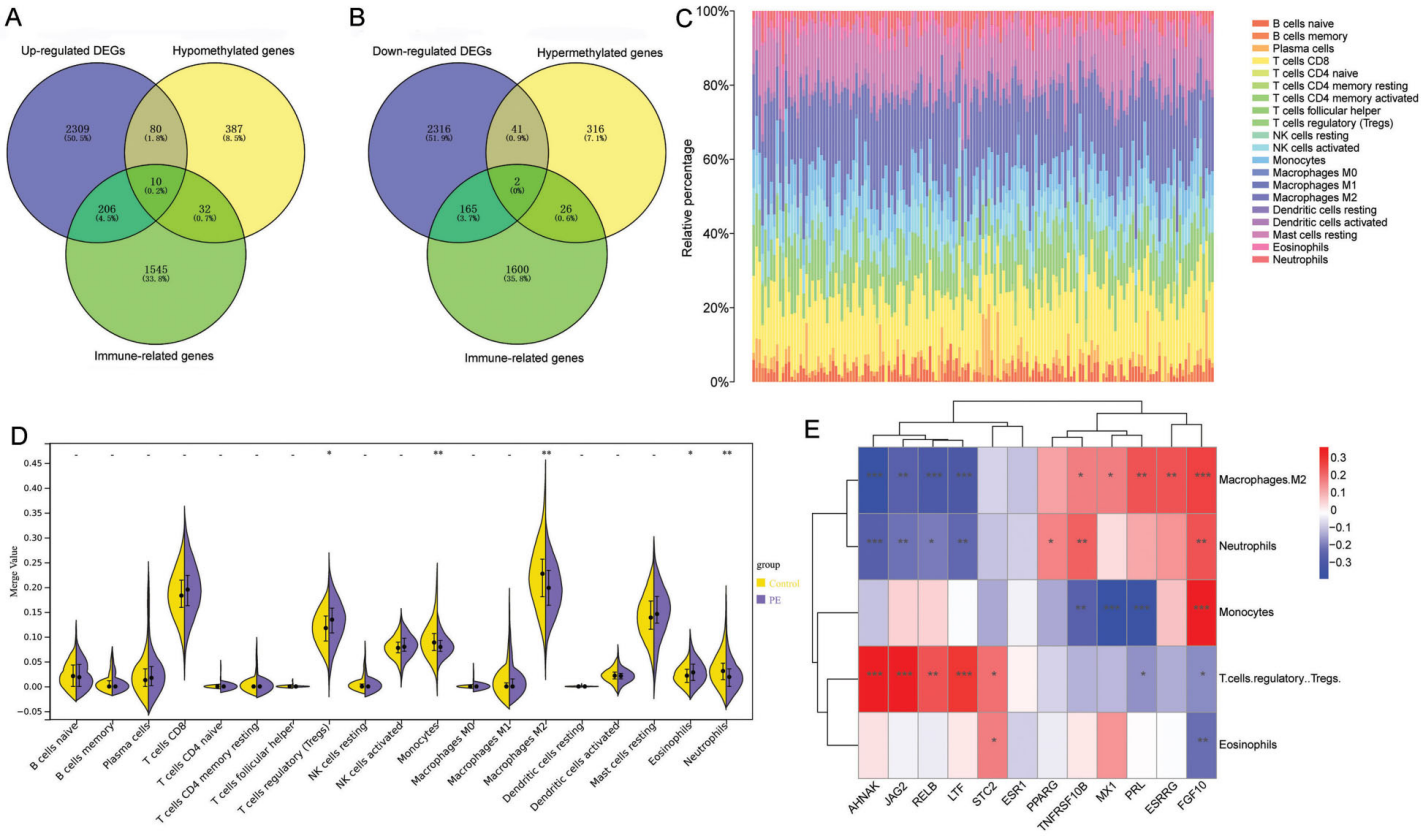
Identification of abnormally methylated immune-related differentially expressed genes (DEGs) and analysis of immune cell infiltration. **A**, Venn diagrams of up-regulated DEGs, hypomethylated genes, and immune-related genes. **B**, Venn diagrams of down-regulated DEGs, hypermethylated genes, and immune-related genes. **C**, Stacked histogram of the proportion of each immune cell in the sample. **D**, Difference of immune cell infiltration between control and preeclampsia (PE) groups. Data are reported as median and interquartile range; Wilcoxon test. **E**, Pearson correlation between differentially infiltrated immune cells and abnormally methylated immune-related DEGs. *P<0.05; **P<0.01; ***P<0.001.

A total of 12 abnormally methylated immune-related DEGs were obtained through the intersection of DEGs, abnormally methylated genes, and immune-related genes (Figure 3A and B). Among them, there were 10 hypomethylated-up-regulated immune-related DEGs (*AHNAK*, *STC2*, *PPARG*, *LTF*, *MX1*, *ESR1*, *RELB*, *JAG2*, *PRL*, and *TNFRSF10B*) and 2 hypermethylated-down-regulated immune-related DEGs (*ESRRG* and *FGF10*).

The proportion of immune cells in the sample was evaluated using the CIBERSORT method to obtain immune cell scores (Figure 3C). Then, immune cell scores were arranged into an immune cell infiltration matrix to examine the differences of immune cell infiltration levels between the control and the PE groups. The results showed that the infiltration levels of monocytes, M2 macrophages, and neutrophils were significantly decreased, and the infiltration levels of regulatory T cells (Tregs) and eosinophils were significantly increased in the PE group (Figure 3D). Pearson correlation analysis showed that *FGF10* was significantly correlated with monocytes, M2 macrophages, neutrophils, Tregs, and eosinophils (Figure 3E). *AHNAK*, *JAG2*, *RELB*, and *LTF* were significantly positively correlated with Tregs, but significantly negatively correlated with M2 macrophages and neutrophils.

### Identification of key abnormally methylated immune-related DEGs and construction of classification models

**Figure 4:**
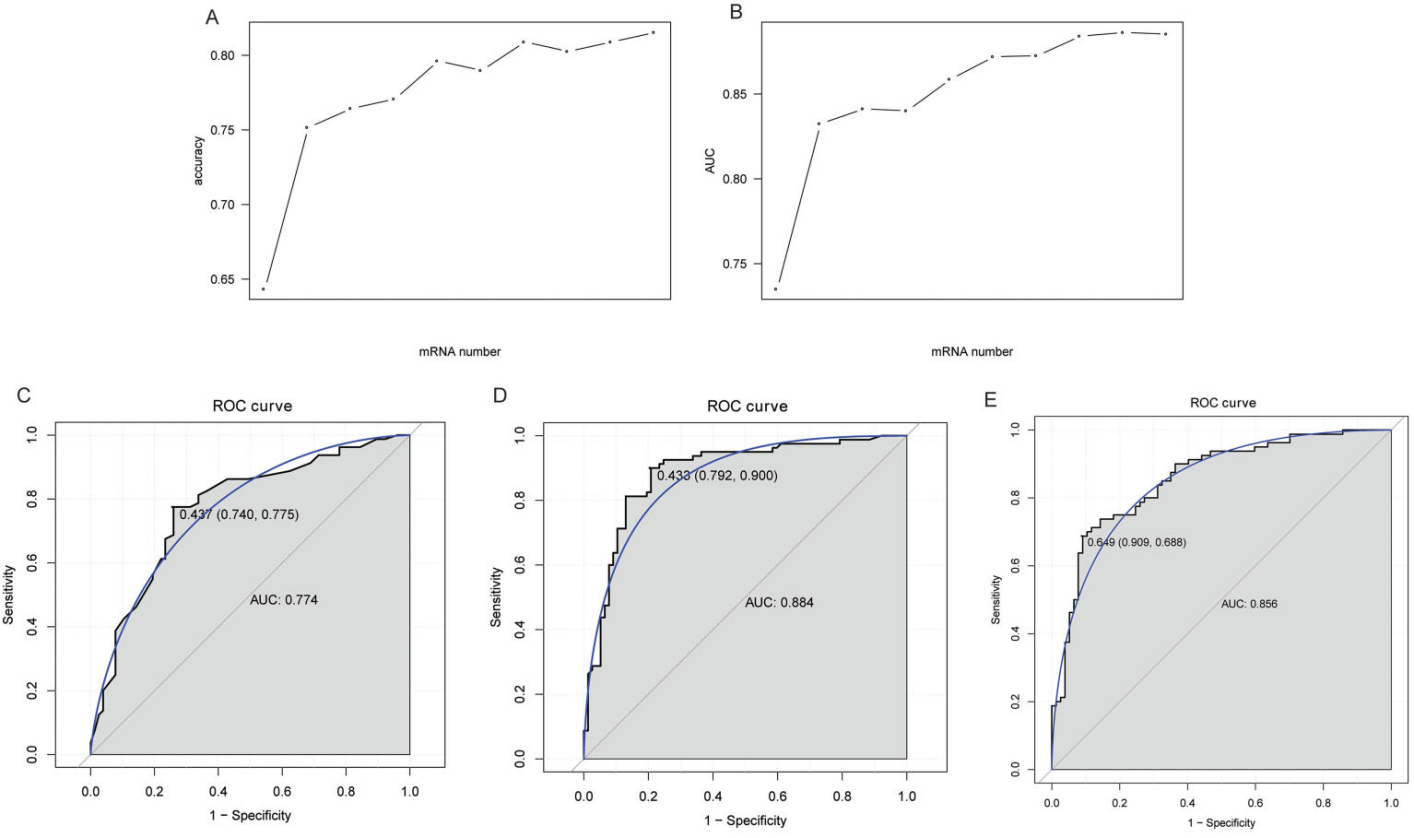
Identification of key abnormally methylated immune-related differentially expressed genes (DEGs) and construction of classification models. **A**, Trend chart of accuracy with the increase of abnormally methylated immune-related DEGs. **B**, Trend chart of AUC with the increase of abnormally methylated immune-related DEGs quantity. **C**, ROC curves of the decision tree (DT) classification model. **D**, ROC curves of the random forests (RF) classification model. **E**, ROC curves of the support vector machine (SVM) classification model.

**Figure 5:**
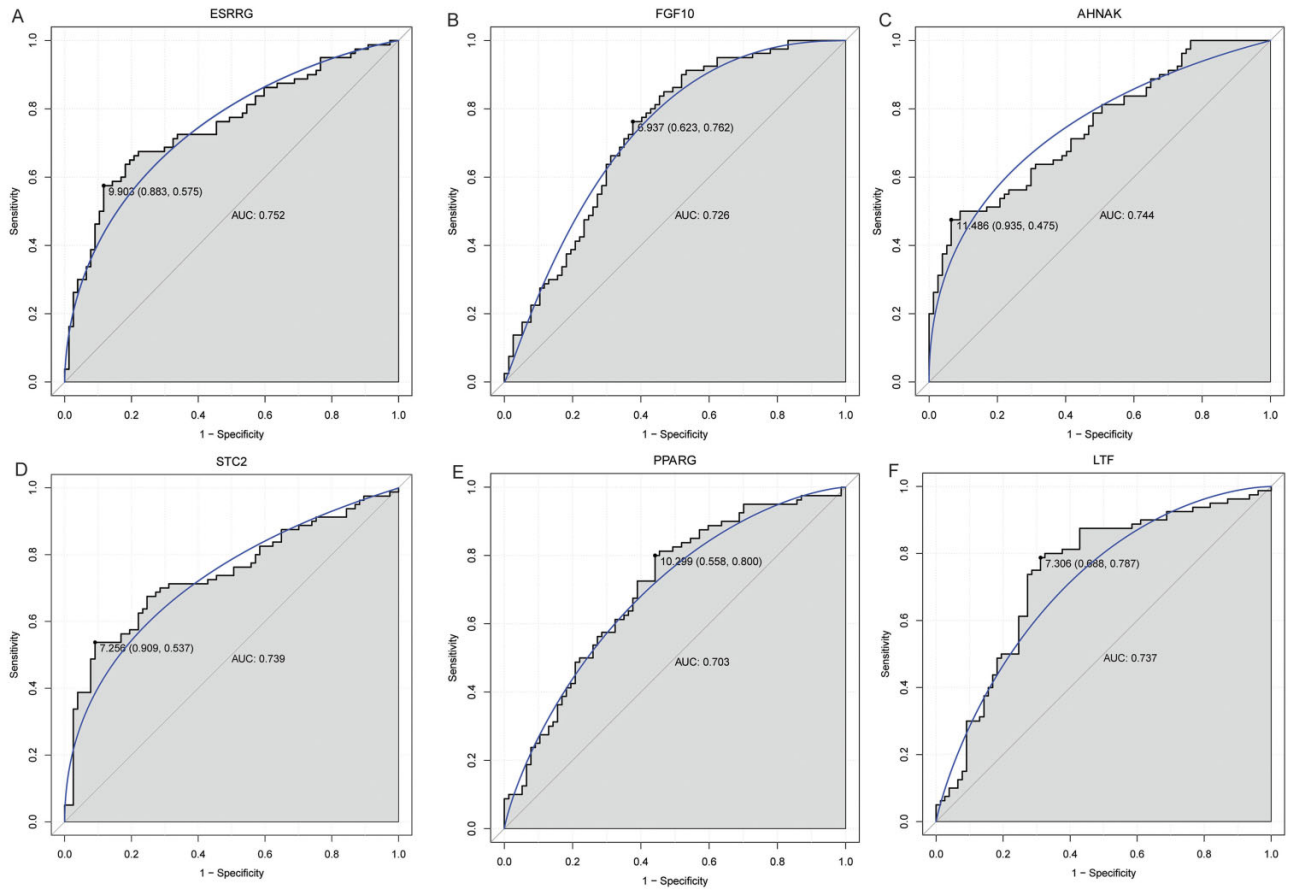
**A-F**, ROC analysis of diagnostic ability of abnormally methylated immune-related differentially expressed genes (DEGs).

Based on AUC values, *ESRRG*, *FGF10*, *AHNAK*, *STC2*, *PPARG*, *LTF*, *MX1*, *ESR1*, *RELB*, and *JAG2* were selected as the key abnormally methylated immune-related DEGs from 12 abnormally methylated immune-related DEGs (Figure 4A and B). Subsequently, DT, RF, and SVM classification models were constructed based on these 10 key abnormally methylated immune-related DEGs. The AUC of the ROC curve of DT (Figure 4C), RF (Figure 4D), and SVM (Figure 4E) were 0.774, 0.884, and 0.856, respectively, indicating that these models have a certain level of diagnostic accuracy. In addition, ROC analysis was also performed for *ESRRG*, *FGF10*, *AHNAK*, *STC2*, *PPARG*, *LTF*, *MX1*, *ESR1*, *RELB*, and *JAG2*. The results showed that the AUC values of *ESRRG*, *FGF10*, *AHNAK*, *STC2*, *PPARG*, and *LTF* ranged from 0.7 to 0.8, indicating that they have some degree of diagnostic accuracy (Figure 5A-F). The AUC values of *MX1*, *ESR1*, *RELB*, and *JAG2* ranged from 0.6 to 0.7 (Supplementary Figure S3). Moreover, we found that the AUC values of single abnormally methylated immune-related DEG were all lower than those of the DT, RF, and SVM diagnostic models. This may suggest that the classification models have relatively higher diagnostic value compared to single DEGs.

### Construction of miRNA-miRNA network

**Figure 6:**
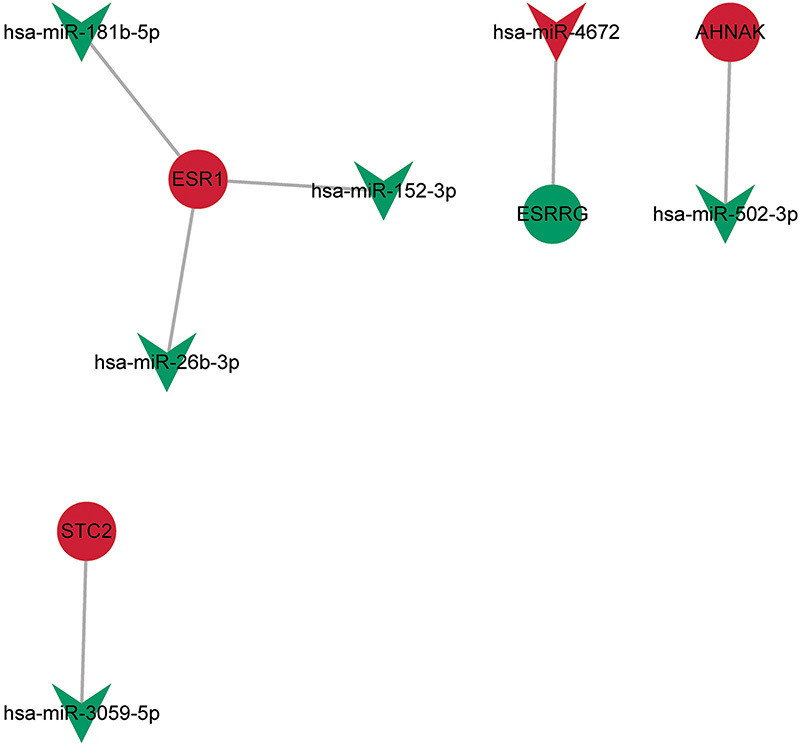
Construction of the miRNA-mRNA network. V-shape and circle represent miRNA and mRNA, respectively. Green and red represent down-regulated and up-regulated, respectively.

In this study, a total of 259 targeted miRNAs were predicted for key abnormally methylated immune-related DEGs based on TargetScan and miRDB databases. Furthermore, based on the P<0.05 screening criterion, 197 DEmiRNAs were identified in the GSE206988 dataset. The predicted miRNAs and DEmiRNA were intersected to obtain negatively regulated miRNA-mRNA targeting pairs. Subsequently, the results were imported into Cytoscape to construct the miRNA-mRNA network (Figure 6). The miRNA-mRNA network included 4 key abnormally methylated immune-related DEGs and 6 targeted DEmiRNAs. A total of 6 miRNA-mRNA relationship pairs were identified, namely hsa-miR-181b-5p-ESR1, hsa-miR-152-3p-ESR1, hsa-miR-26b-3p-ESR1, hsa-miR-4672-ESRRG, hsa-miR-502-3p-AHNAK, and hsa-miR-3059-5p-STC2.

### Expression verification

The GSE10588 dataset was downloaded from the GEO database to verify the expression of key abnormally methylated immune-related DEGs. The results showed that the expression trends of *ESRRG*, *FGF10*, *STC2*, *PPARG*, *LTF*, *MX1*, and *RELB* were consistent with the expression trends in the GSE75010 dataset (Supplementary Figure S4). However, *FGF10*, *STC2*, and *PPARG* lacked statistical significance, which may be due to sample size and heterogeneity. The specific molecular mechanism needs further study.

### Real-time PCR validation of *ESRRG*, *FGF10*, *AHNAK*, *STC2*, and *ESR1*

**Figure 7:**
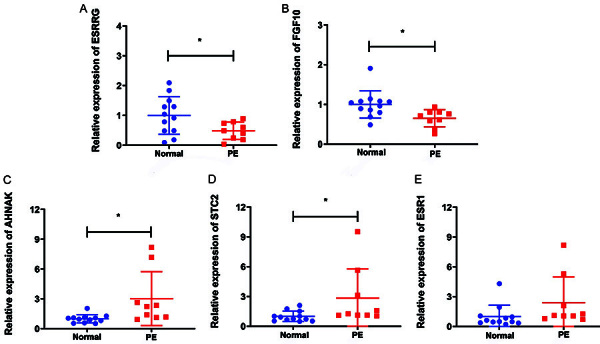
The expression levels of the *ESRRG* (**A**), *FGF10* (**B**), *AHNAK* (**C**), *STC2* (**D**), and *ESR1* (**E**) genes in normal and preeclampsia (PE) samples were verified by real-time PCR. Data are reported as means and SD. *P<0.05. Student’s *t*-test was used for comparisons.

In this study, *ESRRG*, *FGF10*, *AHNAK*, *STC2*, and *ESR1* were randomly selected for expression validation by real-time PCR in 9 PE placental tissue samples and 12 normal control tissue samples. All primers are shown in Table S2. The real-time PCR results showed that *ESRRG* and *FGF10* were up-regulated in PE, while *AHNAK*, *STC2*, and *ESR1* were down-regulated in PE (Figure 7), which was consistent with the bioinformatics results. However, *ESR1* failed to reach statistical significance, which may be due to sample heterogeneity and small sample size. In the future, additional clinical samples will be collected to increase the sample size for more in-depth validation.

## Discussion

In this study, functional annotation results showed that DEGs and abnormally methylated genes were enriched in immune-related pathways, such as rap1, PI3K-Akt, and calcium signaling pathways. These pathways play an important role in PE (14-16). Notably, our study identified that *FGF10* was enriched in the rap1, PI3K-Akt, and calcium signaling pathways. *FGF10* is a key member of the fibroblast growth factor family and is crucial for multi-organ development and tissue homeostasis. It is expressed in decidual cells and cytotrophoblasts of the cytotrophoblast columns in human pregnancy, and it may play an important role in decidual-trophoblast interaction (17). *FGF10* is abnormally expressed in PE and may be a potential biomarker for early-onset PE (18). While previous studies have linked *FGF10* to decidual-trophoblast interaction and early-onset PE diagnosis, none have reported its association with the rap1/PI3K-Akt/calcium signaling axis. It is speculated that *FGF10* may be related to the immune mechanism of PE, given its enrichment in the rap1, PI3K-Akt, and calcium signaling pathways, but the specific mechanism remains to be further studied.

Normal placental development and function are crucial for fetal growth. ESRRG signaling may affect normal placental functions, including trophoblast function, placental angiogenesis, hypoxic responses, and placental metabolism (19). Moreover, *ESRRG* is highly expressed in the normal placenta, but is decreased in PE (19). However, prior studies have not linked its expression to methylation, nor have they validated its potential as a diagnostic marker for PE. Similarly, *AHNAK* is reported to modulate trophoblast proliferation, invasion, and apoptosis via miRNA regulation in PE (20), yet the specific miRNA-mRNA interaction networks and how they intersect with immune pathways remain uncharacterized. *STC2* is primarily localized in syncytiotrophoblasts and invasive cytotrophoblasts of the human placenta. Compared with chronic villi, the expression level of *STC2* is significantly elevated in the placental basal plate, and its role in PE warrants further in-depth investigation (21,22). *PPARG* is crucial for placental development, and changes in its expression and activity are associated with human placental pathologies (23). A previous study showed that *PPARG* is a DEG with abnormally methylated modification and may be a potential diagnostic marker for PE (24). *LTF* is one of the genes highly associated with PE, and its abnormal expression may be involved in the occurrence and development of PE (25). In this study, the AUC values of these 5 key abnormally methylated immune-related DEGs were greater than 0.7, indicating that they have a certain degree of diagnostic accuracy and may be potential diagnostic biomarkers for PE.

MX1, a key antiviral protein involved in immune homeostasis, has been implicated in PE pathogenesis (26,27), but its specific role in the immune dysregulation that drives PE remains unclear. As a member of the nuclear receptor superfamily, *ESR1* is involved in regulating the functions of innate immune cells and related signaling pathways (28). *ESR1* expression is dysregulated in PE and is associated with defective decidualization in severe PE (29,30). To date, no research has reported that *RELB* may be related to the pathological mechanism of PE. *JAG2*, as a key ligand of the Notch signaling pathway, plays an important role in immune regulation (31). *JAG2* exhibits abnormal expression in PE (32), but its specific mechanism of action requires further investigation. In this study, *MX1*, *ESR1*, *RELB*, and *JAG2*, as key abnormally methylated immune-related DEGs, may be associated with the pathological mechanism of PE. The specific molecular mechanisms deserve further research.

This study preliminarily explored the characteristics of the immune microenvironment in PE patients using the CIBERSORT method. Compared with the control group, the infiltration levels of monocytes, M2 macrophages, neutrophils, Tregs, and eosinophils in the PE group were abnormal. The existing literature indicates that monocytes, M2 macrophages, neutrophils, and Tregs are important cell subsets involved in the pathogenesis of PE (33,34). Pearson correlation analysis revealed that the key abnormally methylated immune-related DEGs (except *ESR1*) were significantly correlated with these immune cells, suggesting that the identified key abnormally methylated immune-related DEGs may be related to the abnormal infiltration of these immune cells.

To further understand the molecular mechanisms of key abnormally methylated immune-related DEGs, the miRNA-miRNA network was also constructed, which identified 6 miRNA-mRNA relationship pairs. Of those, hsa-miR-181b-5p can affect the migration and invasion of trophoblast cells (35). A study has shown that the signaling axis formed by hsa-miR-181b-5p and RBAK can be regulated by puerarin to alleviate PE-induced inhibition of trophoblast cell activity and inflammatory response (36). An *in vitro* study showed that hsa-miR-26b-3p may mediate the occurrence and progression of PE by regulating the proliferation, invasion, and apoptosis of trophoblast cells (37). Hsa-miR-152-3p is also abnormally expressed in PE (38). Our research findings suggest that hsa-miR-181b-5p, hsa-miR-26b-3p, and hsa-miR-152-3p are also abnormally expressed in PE. Furthermore, these miRNAs were associated with ESR1, providing a reasonable mechanistic basis for their dysregulation in PE. To date, there have been no reports on the association of hsa-miR-4672, hsa-miR-502-3p, and hsa-miR-3059 and PE. This study is the first to identify that hsa-miR-4672, hsa-miR-502-3p, and hsa-miR-3059 exhibit abnormal expression in PE. The specific mechanism of action of the identified miRNA-mRNA relationship pairs in PE remains unclear, and further research is required.

Constructing classification diagnostic models based on genes has unique advantages in medical disease research. Diseases are usually driven by multi-gene regulatory networks, and the diagnostic efficacy of a single biomarker is limited. Machine learning models can integrate the interactions among multiple genes to improve diagnostic accuracy. In this study, DT, RF, and SVM classification models were constructed based on 10 key abnormally methylated immune-related DEGs. The classification models based on these 10 genes exhibited a medium level of diagnostic accuracy. Moreover, compared with a single DEG, these classification models may have relatively higher potential diagnostic reference value. Future research should further validate these classification models in larger cohorts and explore their clinical utility in early PE detection and personalized management strategies.

This study had some limitations. Firstly, the specific mechanisms by which identified mRNA-miRNA pairs and key abnormally methylated immune-related DEGs modulate relevant signaling pathways and immune cells remain unclear. Therefore, extensive *in vivo* and *in vitro* studies are required to explore their roles and mechanisms in PE. Secondly, the data used to construct the classification models in this study were from a public database, without external independent validation. Therefore, a large number of clinical samples need to be collected to verify the classification models. Thirdly, in the real-time PCR validation, one of the five tested genes failed to reach statistical significance, which may be due to sample heterogeneity and small sample size. In the future, additional clinical samples will be collected to increase the sample size for more in-depth validation. Fourthly, the failure to validate the methylation status of the identified key abnormally methylated genes in clinical samples is another limitation of this study, and we plan to conduct supplementary validation for this in subsequent research.

In summary, the diagnostic models constructed in this study provide a new strategy for the accurate diagnosis of PE. Moreover, it reveals the potential role of abnormally methylated immune-related DEGs in the immune mechanism of PE, laying a foundation for subsequent molecular mechanism research and clinical translation.

## Data Availability

The datasets analyzed during the current research are available in the GEO database (https://www.ncbi.nlm.nih.gov/geo/). Accession numbers of the datasets used in the current study are GSE75010, GSE75196, and GSE206988. All data generated or analyzed during this study are included in this published article.

## Supplementary Materials

Supplementary Material Click to view [pdf].

## References

[1] Ma'ayeh M, Costantine MM (2020). Prevention of preeclampsia. Semin Fetal Neonatal Med.

[2] Bellamy L, Casas JP, Hingorani AD, Williams DJ (2007). Pre-eclampsia and risk of cardiovascular disease and cancer in later life: systematic review and meta-analysis. BMJ.

[3] Gathiram P, Moodley J (2016). Pre-eclampsia: its pathogenesis and pathophysiolgy. Cardiovasc J Afr.

[4] Saito S, Shiozaki A, Nakashima A, Sakai M, Sasaki Y (2007). The role of the immune system in preeclampsia. Mol Aspects Med.

[5] Laresgoiti-Servitje E (2013). A leading role for the immune system in the pathophysiology of preeclampsia. J Leukoc Biol.

[6] Rani BU, Vasantharekha R, Santosh W, Swarnalingam T, Barathi S (2025). Endocrine-disrupting chemicals and the effects of distorted epigenetics on preeclampsia: a systematic review. Cells.

[7] Choudhury M, Friedman JE (2012). Epigenetics and microRNAs in preeclampsia. Clin Exp Hypertens.

[8] Deng F, Huang J, Yuan X, Cheng C, Zhang L (2021). Performance and efficiency of machine learning algorithms for analyzing rectangular biomedical data. Lab Invest.

[9] Šimundić AM (2009). Measures of diagnostic accuracy: basic definitions. EJIFCC.

[10] Leavey K, Benton SJ, Grynspan D, Kingdom JC, Bainbridge SA, Cox BJ (2016). Unsupervised placental gene expression profiling identifies clinically relevant subclasses of human preeclampsia. Hypertension.

[11] Yeung KR, Chiu CL, Pidsley R, Makris A, Hennessy A, Lind JM (2016). DNA methylation profiles in preeclampsia and healthy control placentas. Am J Physiol Heart Circ Physiol.

[12] Fan Y, Han Q, Li J, Ye G, Zhang X, Xu T (2022). Revealing potential diagnostic gene biomarkers of septic shock based on machine learning analysis. BMC Infect Dis.

[13] Wang Y, Chen L, Ju L, Xiao Y, Wang X (2020). Tumor mutational burden related classifier is predictive of response to PD-L1 blockade in locally advanced and metastatic urothelial carcinoma. Int Immunopharmacol.

[14] Yang P, Liu X, Lyu J, Feng Q, Ding Y, Zhong S (2025). Down-regulation of TAGLN2 associated with the development of preeclampsia by effecting the Rap1 signaling pathway. Placenta.

[B15] Ye L, Huang Y, Liu X, Zhang X, Cao Y, Kong X (2023). Apelin/APJ system protects placental trophoblasts from hypoxia-induced oxidative stress through activating PI3K/Akt signaling pathway in preeclampsia. Free Radic Biol Med.

[16] Jiang R, Teng Y, Huang Y, Gu J, Ma L, Li M (2014). Preeclampsia serum-induced collagen I expression and intracellular calcium levels in arterial smooth muscle cells are mediated by the PLC-γ1 pathway. Exp Mol Med.

[17] Anteby EY, Natanson-Yaron S, Hamani Y, Sciaki Y, Goldman-Wohl D, Greenfield C (2005). Fibroblast growth factor-10 and fibroblast growth factor receptors 1-4: expression and peptide localization in human decidua and placenta. Eur J Obstet Gynecol Reprod Biol.

[18] Palanisamy TB, Arumugam M (2025). Transcriptomic analysis reveals potential biomarkers for early-onset pre-eclampsia using integrative bioinformatics and LASSO based approach. Comput Biol Med.

[19] Zou Z, Forbes K, Harris LK, Heazell AEP (2021). The potential role of the E SRRG pathway in placental dysfunction. Reproduction.

[20] Shan Z, Zhou L, Ma Y, Huang Y (2025). Circ_0090100 induces AHNAK expression to inhibit trophoblast cell proliferation and invasion and accelerate cell apoptosis by segregating miR-139-5p in preeclampsia. Hum Cell.

[21] Kjaer ASL, Sehgal S, Wu Y, Iyer P, Winn VD (2023). Placental expression of stanniocalcin 2 (STC2) in healthy and preeclamptic pregnancies. Am J Obstetr Gynecol.

[22] Khatun M, Modhukur V, Piltonen TT, Tapanainen JS, Salumets A (2024). Stanniocalcin protein expression in female reproductive organs: literature review and public cancer database analysis. Endocrinology.

[23] Shoaito H, Chauveau S, Gosseaume C, Bourguet W, Vigouroux C, Vatier C (2020). Peroxisome proliferator-activated receptor gamma-ligand-binding domain mutations associated with familial partial lipodystrophy type 3 disrupt human trophoblast fusion and fibroblast migration. J Cell Mol Med.

[24] Jiang L, Chang R, Liu J, Xin H (2022). Methylation-based epigenetic studies and gene integration analysis of preeclampsia. Ann Transl Med.

[25] Brew O, Sullivan MH, Woodman A (2016). Comparison of normal and pre-eclamptic placental gene expression: a systematic review with meta-analysis. PLoS One.

[26] Haller O, Staeheli P, Schwemmle M, Kochs G (2015). Mx GTPases: dynamin-like antiviral machines of innate immunity. Trends Microbiol.

[27] Shabani M, Eghbali M, Abiri A, Abiri M (2024). Comprehensive microarray analysis of severe preeclampsia placenta to identify differentially expressed genes, biological pathways, hub genes, and their related non-coding RNAs. Placenta.

[28] Kovats S (2015). Estrogen receptors regulate innate immune cells and signaling pathways. Cell Immunol.

[29] Gomes VCL, Gilbert BM, Bernal C, Crissman KR, Sones JL (2024). Estrogen and progesterone receptors are dysregulated at the BPH/5 mouse preeclamptic-like maternal-fetal interface. Bilology (Basel).

[30] Garrido-Gomez T, Castillo-Marco N (2021). Disrupted PGR-B and ESR1 signaling underlies defective decidualization linked to severe preeclampsia. Elife.

[31] Kijima M, Yamaguchi T, Ishifune C, Maekawa Y, Koyanagi A, Yagita H (2008). Dendritic cell-mediated NK cell activation is controlled by Jagged2-Notch interaction. Proc Natl Acad Sci USA.

[32] Fragkiadaki P, Soulitzis N, Sifakis S, Koutroulakis D, Gourvas V, Vrachnis N (2015). Downregulation of notch signaling pathway in late preterm and term placentas from pregnancies complicated by preeclampsia. PLoS One.

[33] Miller D, Motomura K, Galaz J, Gershater M, Lee ED, Romero R (2022). Cellular immune responses in the pathophysiology of preeclampsia. J Leukoc Biol.

[34] Jiang P, Zhu X, Jiang Y, Li H, Luo Q (2024). Targeting JUNB to modulate M2 macrophage polarization in preeclampsia. Biochim Biophys Acta Mol Basis Dis.

[35] Miao J, Zhu Y, Xu L, Huang X, Zhou X (2020). miR-181b-5p inhibits trophoblast cell migration and invasion through targeting S1PR1 in multiple abnormal trophoblast invasion-related events. Mol Med Rep.

[36] Guo J, Bian W, Jiang H (2022). Puerarin attenuates preeclampsia-induced trophoblast mobility loss and inflammation by modulating miR-181b-5p/RBAK axis. Am J Reprod Immunol.

[37] Li Y, Ma X, Wu X, Long L (2020). MicroRNA-26b-3p inhibits human trophoblast cell proliferation, invasion and resistance to apoptosis via targeting SHBG. J King Saud Univ Sci.

[38] Sumawan H, Giantari I, Mubarika S, Hadiati DR, Pradjatmo H (2024). Exosomal miRNAs as potential biomarkers for preeclampsia: miR-1283 has the highest expression, while miR-152-3p has the lowest expression. Indon Biomed J.

