# Supplementary material for: Unveiling preeclampsia: diagnostic value and potential molecular mechanisms of abnormally methylated immune-related genes
Source: Braz J Med Biol Res. 2026 Mar 9;59:e15317. doi: 10.1590/1414-431X2025e15317 (PMC12971016; doi:10.1590/1414-431X2025e15317)
Supplement: Supplementary Material [file 1414-431X-bjmbr-59-e15317-suppl.pdf]

Figure S1. Volcano map and heat map of DEGs. **A**, Volcano map of DEGs between the control and PE groups. **B**, Heat map of DEGs between the control and PE groups. Red and blue represent up-regulated and down-regulated, respectively. DEGs, differentially expressed genes; PE, preeclampsia.

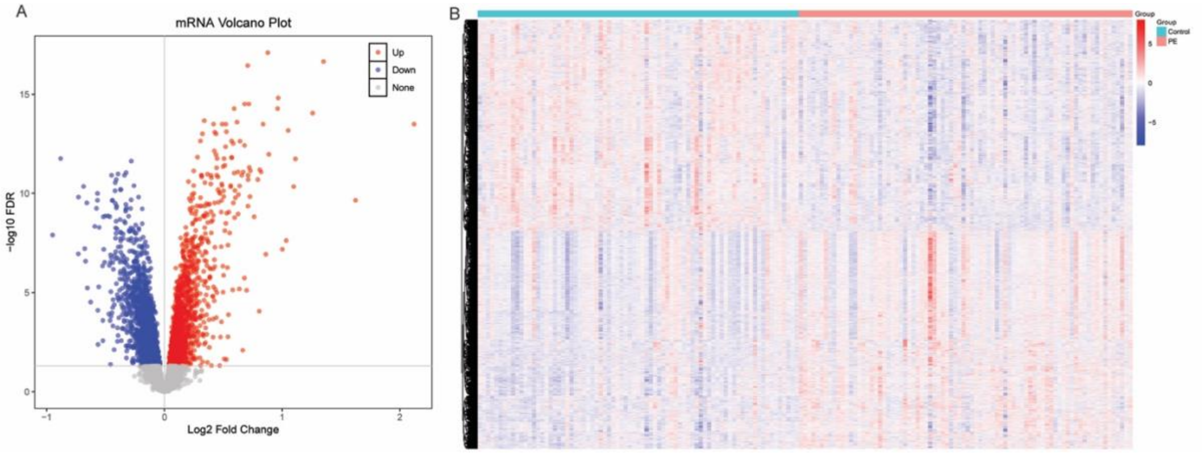

Figure S2. Volcano map and Manhattan map of methylation sites. **A**, Volcano map of differential methylation sites between the control and preeclampsia (PE) groups. Red and blue represent up-regulated and down-regulated, respectively. **B**, Manhattan map of methylation sites.

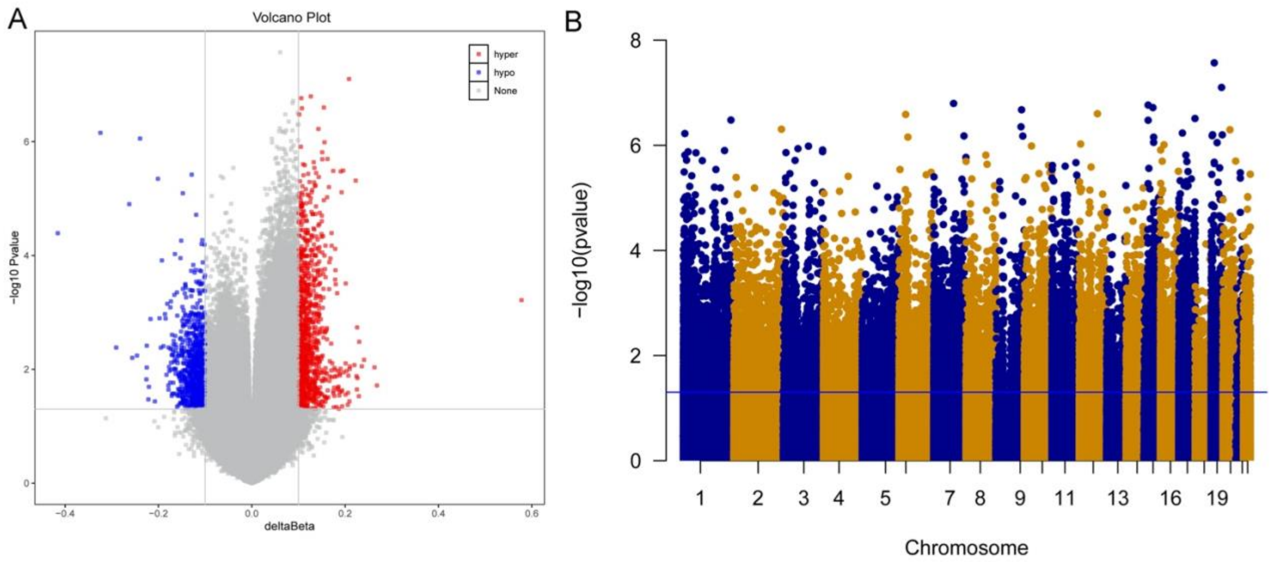

Figure S3. ROC analysis of *MX1* (**A**), *ESR1* (**B**), *RELB* (**C**), and *JAG2* (**D**).

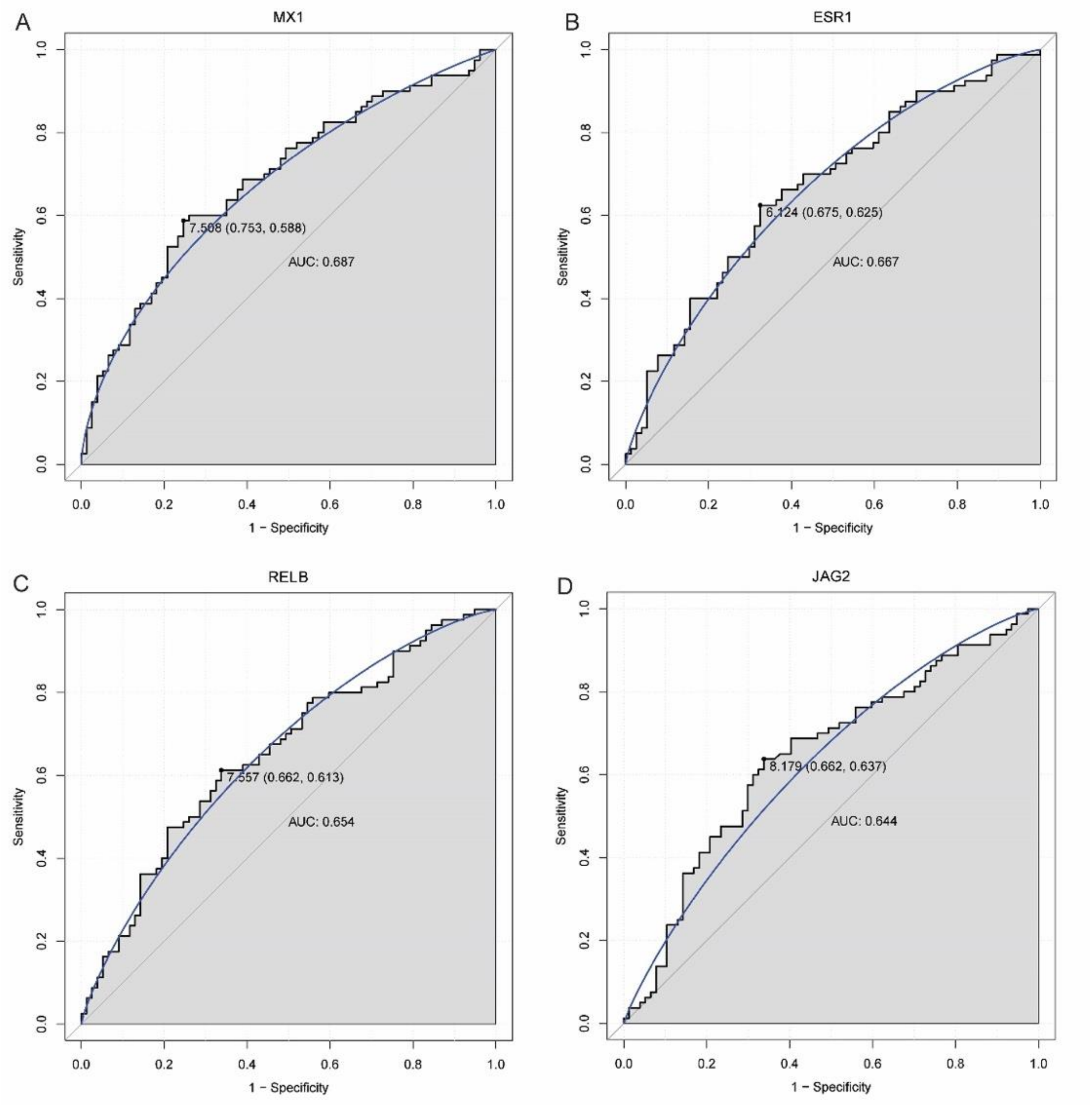

Figure S4. Expression verification of *ESRRG* (**A**), *FGF10* (**B**), *STC2* (**C**), *PPARG* (**D**), *LTF* (**E**), *MX1* (**F**), and *RELB* (**G**) was conducted in the GSE10588 dataset. *P<0.05; **P <0.01; ****P<0.0001; ns: no significant difference (Wilcoxon test).

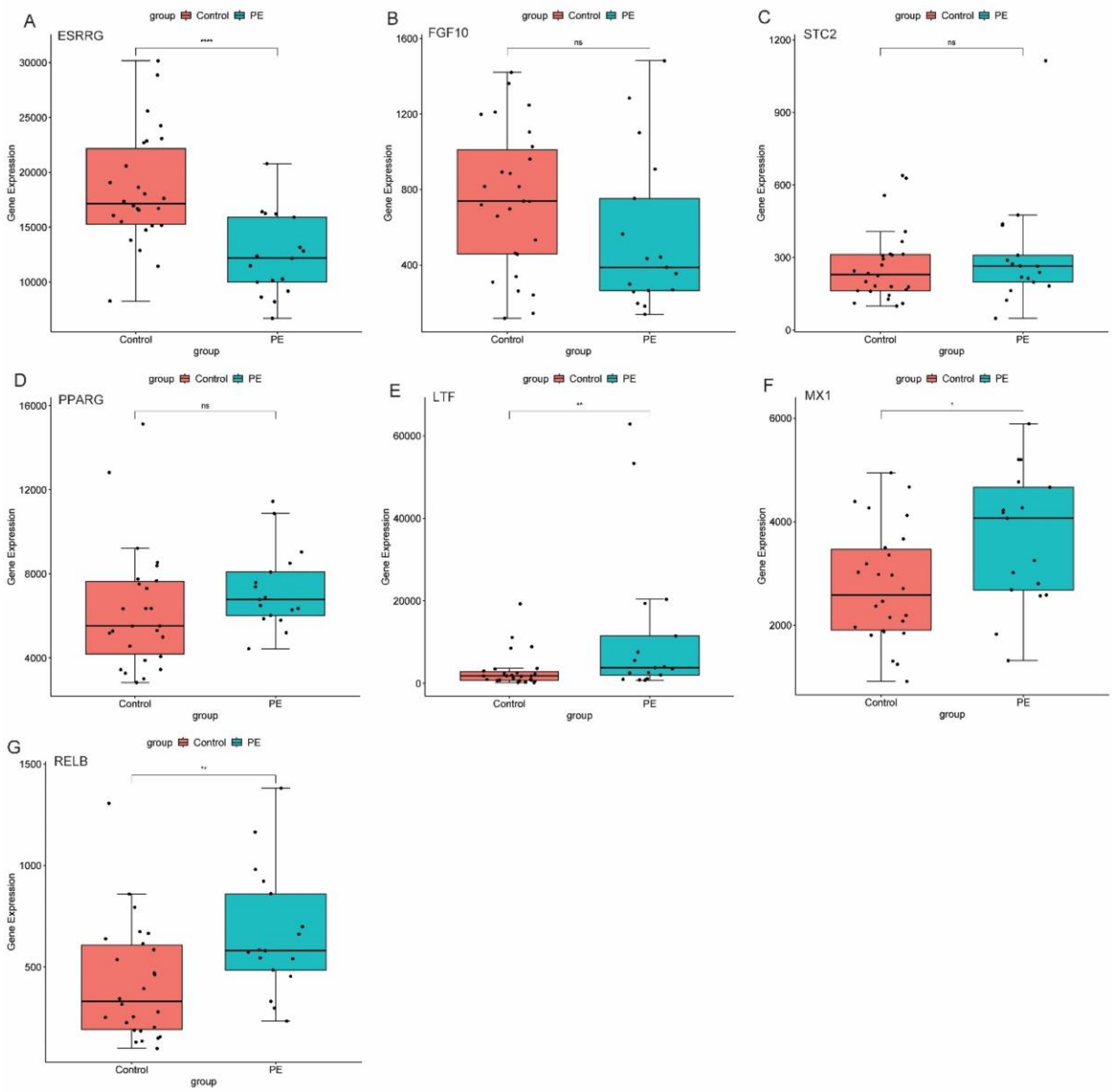

Table S1. Details of the GSE75010 and GSE75196 datasets used to compare preeclampsia (PE) and control samples.

GEO accession	Data type	Author	Platform	Samples (control:PE)	Year	Tissue
GSE75010	mRNA transcriptome data	Leavey K	GPL6244 [HuGene-1_0-st] Affymetrix Human Gene 1.0 ST Array [transcript (gene) version]	77:80	2016	Placental
GSE75196	DNA methylation data	Lind J	GPL13534 Illumina HumanMethylation450 BeadChip (HumanMethylation450_15017482)	16:8	2016	Placental

Table S2. Primer sequences used for real-time PCR validation.

Primer name	Primer sequence (5' to 3')
GAPDH-F (Internal reference)	5-GGAGCGAGATCCCTCCAAAAT-3
GAPDH-R (Internal reference)	5-GGCTGTTGTCATACTTCTCATGG-3
ACTB-F (Internal reference)	5-CATGTACGTTGCTATCCAGGC-3
ACTB-R (Internal reference)	5-CTCCTTAATGTCACGCACGAT-3
ESRRG-F	5-AGGTCGGCAGAAGTACAAGC-3
ESRRG-R	5-TGCTTCGCCCATCCAATGAT-3
FGF10-F	5-CAGTAGAAATCGGAGTTGTTGCC-3
FGF10-R	5-TGAGCCATAGAGTTTCCCCTTC-3
AHNAK-F	5-TACCCTTCCTAAGGCTGACATT-3
AHNAK-R	5-TTGGACCCTTGAGTTTTGCAT-3
STC2-F	5-ACAGGTTCGGCTGCATAAGC-3
STC2-R	5-GAGGTCCACGTAGGGTTCG-3
ESR1-F	5-GGGAAGTATGGCTATGGAATCTG-3
ESR1-R	5-TGGCTGGACACATATAGTCGTT-3

F: forward; R: reverse.
